# The impact of technology on treatment of iatrogenic cervicothoracic vascular traumas: a study of two cases three decades apart and a review of the literature

**DOI:** 10.1590/1677-5449.005618

**Published:** 2018

**Authors:** Victor Bilman, Bernardo Massière, Alberto Vescovi, Daniel Leal, Paula Vivas, Bruno Demier, Arno von Ristow

**Affiliations:** 1 Pontifícia Universidade Católica do Rio de Janeiro – PUC-Rio, Cirurgia Vascular e Endovascular, Rio de Janeiro, RJ, Brasil.; 2 Centro Integrado para a Pesquisa, Prevenção, Diagnóstico e Terapia das Doenças Vasculares – CENTERVASC, Rio de Janeiro, RJ, Brasil.; 3 Academia Nacional de Medicina – ANM, Rio de Janeiro, RJ, Brasil.

**Keywords:** aneurysm, false, vertebral artery, central venous access, endovascular procedures, self-expanding metallic stents

## Abstract

Complications such as pseudoaneurysms (PA) related to cervicothoracic venous access can be devastating. In this article, we present two similar cases in which technological advances impacted diagnosis, treatment, and results. Both patients developed massive PA after deep venous puncture attempts. The first case occurred in 1993 and was diagnosed by a duplex scan that revealed a large PA originating from the right subclavian artery. The artery was approached by median sternotomy with supraclavicular extension. The PA originated from the thyrocervical trunk and was treated with simple ligation. The second case was in 2017. Angiotomography revealed a PA originating in the vertebral artery, which was treated with endovascular techniques, maintaining vessel patency. Both patients progressed satisfactorily, despite quite different approaches. Cervicothoracic vascular lesions represent a diagnostic and therapeutic challenge, where the risk of rupture is high. Technological advances have reduced the risks involved in management of vascular injuries with difficult surgical access.

## INTRODUCTION

 Deep venous accesses have become routine practice in hospital settings. They are essential for many diagnostic and therapeutic procedures. 1 ^-^ 3 Iatrogenic lesions of the subclavian artery and its branches are rare complications, but are associated with serious morbidity and mortality. 4 ^,^ 5 Inamasu et al. and Bernik et al. report incidence rates of post-puncture arterial injuries ranging from 0.4 to 9.9% and 0.5 to 11.4%, respectively, most often involving the common carotid artery. In the majority of cases, symptoms are delayed and the most common complications are arteriovenous fistulas and pseudoaneurysms (PAs). 1 ^,^ 3

 The advent of endovascular surgery has enabled great advances in management of these iatrogenic injuries. While Inamasu et al. describe surgical excision of the PA, with or without arterial reconstruction, as first-line treatment, the complex access needed for this surgery can lead to a series of complications. 1

 This article is a comparative study of two cases involving similar iatrogenic injuries that manifested after puncture for deep venous access and in which different diagnostic and treatment approaches were taken. The objective is to demonstrate how technological advances have influenced treatment of difficult-to-access cervicothoracic vascular traumas. A detailed review of the literature contributed to illustration of the evolution of this subject and supports our conclusions. 

## DESCRIPTION OF THE CASES

**Figure 1:**
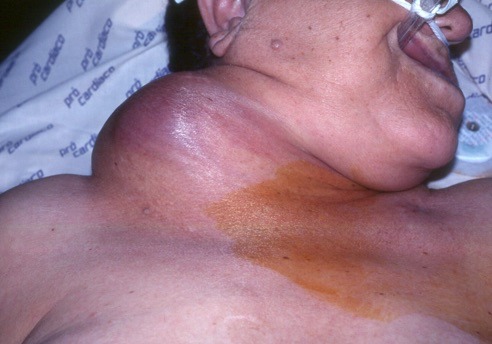
Left cervical mass and hematoma.

**Figure 2:**
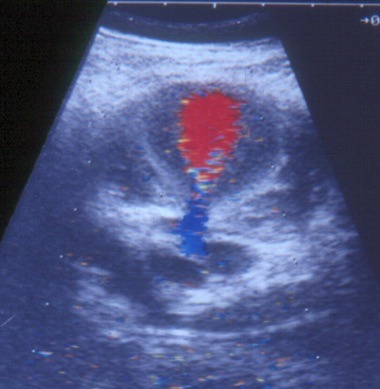
Doppler ultrasonography showing a pseudoaneurysm with turbulent flow.

 The first case, which took place in September 1993, involved a 72-year-old female patient with severe heart disease, arterial hypertension, and grade III obesity who had been admitted to an intensive care unit with sepsis of pulmonary origin. An attempt was made to puncture the right subclavian vein, using a supraclavicular technique, but was unsuccessful. One week later, the vascular surgery team was called to investigate a large pulsating mass in the right cervical region. Physical examination found the patient hemodynamically unstable, with a large, right-side, cervical pulsating mass ( Figure 1 ). A duplex scan revealed a PA originating from the second portion of the right subclavian artery (RSA), with diameters of 50 × 42 mm ( Figure 2 ), and the decision was taken to treat the patient with open surgery. 

**Figure 3:**
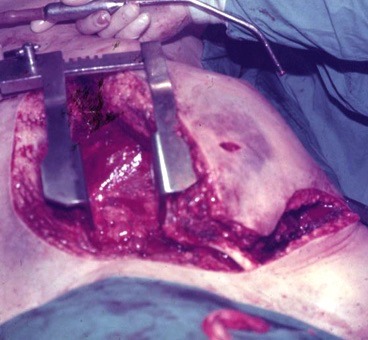
Sternotomy with right supraclavicular extension.

 Access was achieved via a median sternotomy with right supraclavicular extension ( Figure 3 ). The proximal RSA was isolated and the neck of the PA was approached progressively. It originated from the proximal segment of the thyrocervical trunk and was treated with simple ligation. The aneurysm sac was drained. The patient recovered satisfactorily, despite the magnitude of the intervention and the severity of her condition. 

 The second case occurred in December 2017 and involved a 66-year-old female patient with severe heart disease and systemic arterial hypertension who had undergone a kidney transplant in 2007 because of polycystic kidney disease. She was admitted to an intensive care unit with sepsis of pulmonary origin. An attempt was made to perform ultrasound-guided puncture of the left internal jugular vein for administration of vasoactive amines, but the attempt was unsuccessful and the procedure was aborted. The patient developed a pulsating mass in the left cervical region and exhibited a progressive drop in hematocrit levels. After 15 days, during which the patient was in pain and the cervical mass expanded, the vascular surgery team was asked to investigate. 

**Figure 4:**
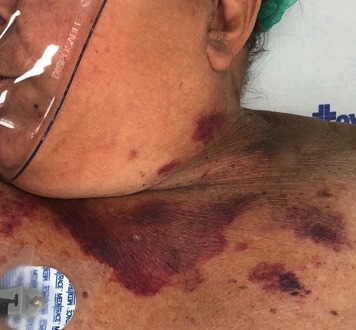
Left cervical mass and hematoma.

**Figure 5:**
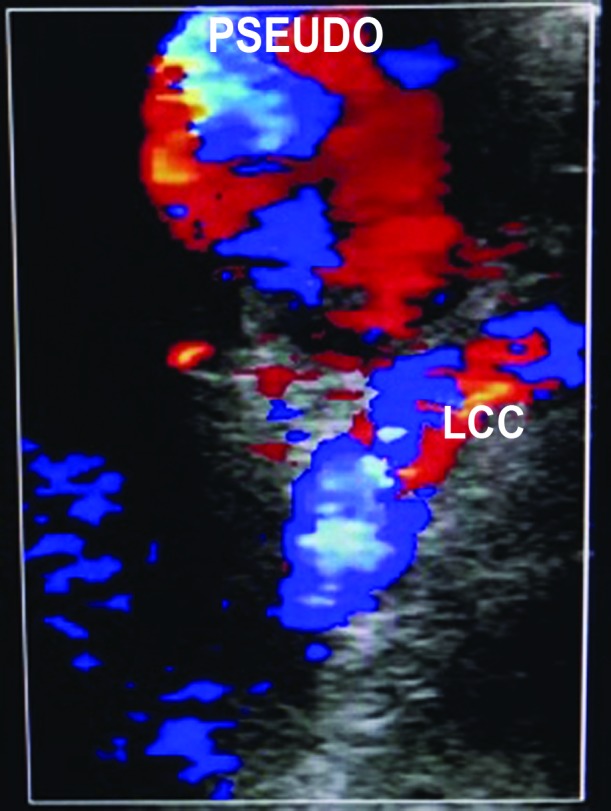
Doppler ultrasonography showing pseudoaneurysm (with suspected origin at the level of the left common carotid artery – LCC) with turbulent flow.

**Figure 6:**
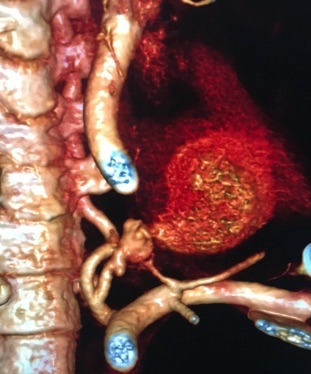
3D angiotomography reconstruction of the left vertebral artery, with no flow filling the pseudoaneurysm.

 During physical examination and history taking, the patient complained of considerable pain in the left cervical region, related to the pulsating mass ( Figure 4 ). A duplex scan suggested a PA originating from the left common carotid artery ( Figure 5 ), and computed tomography angiography (CTA) revealed a PA from segment V1 of the left vertebral artery, with diameters of 30 x 32 mm ( Figure 6 ). 

**Figure 7:**
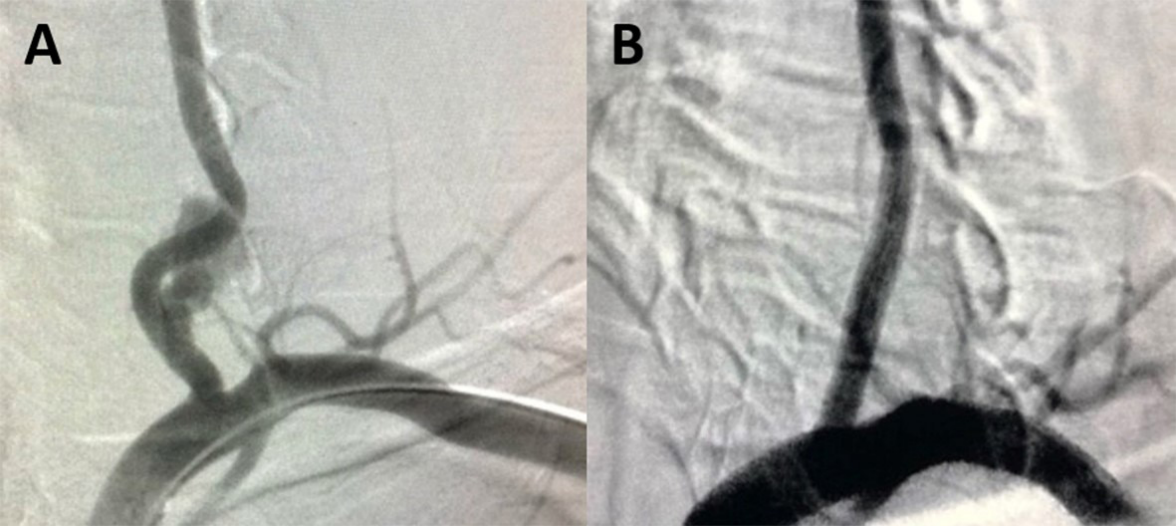
(A) Perioperative arteriography of the left vertebral artery. (B) Control arteriography after placement of a 5 mm × 2.5 cm Viabahn® stent (WL Gore, Flagstaff, AZ, United States).

 After the team had discussed the case, the decision was taken to employ endovascular techniques to implant a Viabahn® 5 mm × 2.5 cm covered stent (WL Gore, Flagstaff, AZ, United States) in the vertebral artery. The procedure was accomplished with no intercurrent conditions and 18 mL of iodinated contrast were used ( Figure 7 ). The cervical mass receded and the pain resolved during the immediate postoperative period and the patient suffered no neurological deficits. A control duplex scan conducted 6 months after the procedure showed that the left vertebral artery was patent at the level of segment V3. 

## DISCUSSION

 Vascular complications related to percutaneous procedures in the cervicothoracic region can be devastating. 4 Iatrogenic traumas to carotid and subclavian arteries can provoke intense bleeding, hematoma, dissection, emboli, or thrombosis, and cases of airway obstruction secondary to expanding cervical hematomas, shock from hemothorax, stroke caused by emboli, PA, and arteriovenous fistula have all been described in the medical literature. 4 Pseudoaneurysm is probably the rarest complication of vertebral artery injuries and there are few cases described in the literature. 3 ^,^ 5 ^-^ 8 Incidence is 0.2%, as reported by Elias et al. 2

 Pseudoaneurysms of the vertebral artery can be caused by penetrating or blunt traumas to the cervical region, arterial dissection, and surgical procedures. Penetrating injuries primarily involve the first portion of the artery, before it enters through the transverse foramen, whereas blunt cervical and cranial traumas involve more distal portions. 2 ^,^ 7 Rapid identification and management of these lesions is fundamental for a satisfactory result, although management is not fully understood and rarely described in the literature. 1 ^,^ 2 While spontaneous resolution of these PAs has been described, rupture is observed in 31 to 54% of patients. 4 ^,^ 9

 In the majority of cases, pseudoaneurysms appear after a delay and, primarily, present as a pulsating mass, with localized pain, dyspnea, heart failure, and cerebral and spinal ischemia. 1 ^-^ 3 Ambekar et al. describe diagnosis after 2 days, Elias et al., after 3 days, Bernik et al., after 4 days, Amaral et al., after 15 days, and Cihangiroglu describe diagnosis after 32 days. 2 ^,^ 3 ^,^ 5 ^,^ 7 ^,^ 10

 The initial diagnostic approach described in the literature is duplex scanning, which shows hematoma with arterial flow through the interior. Although this is a good method, it may be difficult to see the origin of the PA, as pointed out by Cihangiroglu et al. 3 ^,^ 7 Proximity to the internal carotid artery, the volume of the hematoma, and turgidity of the internal jugular vein are factors that can make it difficult to see the origin of the PA. Computed tomography arteriography is superior for viewing the lesion, including its origin, and for correctly identifying the site, the presence of any additional vascular traumas, and the patency of the contralateral vertebral artery, and it is also helpful for planning treatment. 2 ^,^ 3 ^,^ 7

 Treatments for PA include endovascular embolization, direct repair of the vertebral artery or resection of the PA with ligature. 2 ^,^ 7 Bernik et al. also mention ultrasound-guided compression and percutaneous thrombin injection as alternative options. 3 It should be pointed out that the depth of the lesion ruled out treatment with compression and because of the risk of cerebral embolization, treatment with thrombin is not recommended. 3 Aoki et al. describe the case of a patient treated with conservative management who, after 70 days, developed pain and paresthesia in the right shoulder and arm caused by compression of the brachial plexus by hematoma. The PA ruptured and a direct repair was performed during an emergency operation, but the patient died 11 days later. 8

 Amaral et al. suggest open treatment as the first line option when the lesion is symptomatic and located at the origin of the vertebral artery. 10 A median sternotomy, with supraclavicular or transclavicular extension is the best option for proximal control in cases with large right-side hematomas. 3 ^,^ 7 ^,^ 10 Another access option is a single incision along the sternocleidomastoid muscle or a single transclavicular incision, combined with temporary placement of a balloon at the origin of the vertebral artery. 10 Bernik et al. stress the need for excellent proximal control to provide a good view of the lesion and surgical treatment. 3 On the left, access requires an anterior thoracotomy for proximal control of the subclavian artery, since its posterior position means that sternotomy does not offer good control. 11 All of these approaches are associated with elevated morbidity. 11 ^,^ 12

 According to the literature, endovascular treatment offers good results. 4 ^,^ 13 ^-^ 16 This option has changed management of these injuries, which previously was often achieved by ligature, to strategies for exclusion of the PA, maintaining the vessel patent. Ambekar et al. and Kerolus et al. describe using embolization devices such as the Pipeline® (Covidien Vascular Therapies, Mansfield, MA, United States), which divert the flow, preserving vessel patency. In a systematic review, they observed an 82.9% rate of aneurysm elimination at 6 months. 5 Pérez et al. used a chrome-cobalt coated balloon-expandable stent (Papyrus PK Biotronik®) to successfully exclude a vertebral artery PA, maintaining patency of the vessel. 13 In addition to fitting a covered stent, Kwon et al. also successfully embolized a PA using coils. 16 Yamamoto et al. describe using coils and N-butyl cyanoacrylate, resulting in occlusion of the lesion, but also of the vertebral artery. 17 We report, probably for the first time, use of a Viabahn® 5 mm × 2.5 cm covered stent (WL Gore, Flagstaff, AZ, United States) in the vertebral artery, preserving the vessel’s patency. 

 As described by Amaral et al. and Yamamato et al., unilateral occlusion of the vertebral artery is a common practice for treatment of these lesions and, when the contralateral artery is patent, is generally well-tolerated. 10 ^,^ 17 The posterior inferior cerebellar artery on the side of the lesion must be supplied by the contralateral vertebral artery; if this does not occur, then the patient may develop Wallenberg syndrome. 10 This syndrome is characterized by deficit in cranial nerves (V, IX, X, and XI), ataxia, Horner syndrome, and contralateral loss of the ability to feel pain and temperature, among other manifestations of vertebrobasilar insufficiency. It is important to point out that 15% of the population has one dominant vertebral artery, with a hypoplastic contralateral artery, and 5% only have one vertebral artery. 10 Occlusion or ligature of a vertebral artery in non-emergency situations demands confirmation of adequate blood flow to the posterior circulation. 10 ^,^ 18 ^,^ 19

## CONCLUSIONS

 While routine in modern medicine, deep venous punctures are not free from complications. Endovascular treatment has an important role to play in management of patients with iatrogenic vascular traumas. The traditional role of the vascular surgeon is thus being challenged. As is the case with the open repair technique, the vascular surgeon must be acquainted with technological advances, both for workup and for treatment. Endovascular techniques generally offer reduced morbidity and mortality for management of difficult-to-access injuries. 

 While introduction of new technologies, such as ultrasound-guided puncture, has reduced the occurrence of complications related to this procedure, it has not eliminated iatrogenic complications. Even with training and experience, venous punctures are not risk-free. Knowledge of the cervical anatomy and its anatomic variations is as important as judicious use of ultrasonography. Prevention of iatrogenic injuries is the most important point to be taught in hospital settings. 6
