# Supplementary material for: The impact of technology on treatment of iatrogenic cervicothoracic vascular traumas: a study of two cases three decades apart and a review of the literature
Source: J Vasc Bras. 2018 Oct-Dec;17(4):322–7. doi: 10.1590/1677-5449.005618 (PMC6375269; doi:10.1590/1677-5449.005618)
Supplement: Translation

# Translation: O impacto da tecnologia no tratamento do trauma vascular iatrogênico cervicotorácico: estudo de dois casos com intervalo de três décadas e revisão da literatura

**Authors:** Victor Bilman, Bernardo Massière, Alberto Vescovi, Daniel Leal, Paula Vivas, Bruno Demier, Arno von Ristow

**Affiliations:** 1 Pontifícia Universidade Católica do Rio de Janeiro – PUC-Rio, Cirurgia Vascular e Endovascular, Rio de Janeiro, RJ, Brasil.; 2 Centro Integrado para a Pesquisa, Prevenção, Diagnóstico e Terapia das Doenças Vasculares – CENTERVASC, Rio de Janeiro, RJ, Brasil.; 3 Academia Nacional de Medicina – ANM, Rio de Janeiro, RJ, Brasil.

**Keywords:** falso aneurisma, artéria vertebral, cateterismo venoso central, procedimentos endovasculares, stents metálicos autoexpansíveis

## Abstract

Complicações relacionadas ao acesso venoso cervicotorácico, como os pseudoaneurismas (PAs), podem ser devastadoras. Neste artigo, apresentamos dois casos semelhantes em que o avanço tecnológico impactou no diagnóstico, tratamento e resultados. Ambos pacientes apresentaram volumoso PA após a tentativa de punção venosa profunda. O primeiro caso, em 1993, diagnosticado por *duplex scan*, revelou grande PA oriundo da artéria subclávia direita. A artéria foi abordada por esternotomia mediana com extensão supraclavicular. O PA originava-se do tronco tireocervical, tratado com simples ligadura. No segundo caso, em 2017, angiotomografia revelou um PA originário da artéria vertebral, que foi tratado com técnica endovascular, mantendo a perviedade do vaso. Ambos evoluíram satisfatoriamente, apesar de abordagens bastante diferentes. A lesão vascular cervicotorácica representa um desafio propedêutico e terapêutico, com alto risco de ruptura. Os avanços tecnológicos diminuem os riscos de lesões vasculares com acesso cirúrgico difícil e devem estar entre as opções do cirurgião vascular.

## INTRODUÇÃO

 Os acessos venosos profundos tornaram-se prática rotineira nos hospitais. Seu uso é essencial para procedimentos diagnósticos e terapêuticos 1 ^-^ 3 . A lesão iatrogênica da artéria subclávia e seus ramos é uma complicação rara, mas associada a grave morbidade e mortalidade 4 ^,^ 5 . Inamasu et al. e Bernik et al. relatam incidência de lesões arteriais pós-punção variando de 0,4 a 9,9% e 0,5 a 11,4%, respectivamente, mais comumente associadas à artéria carótida comum. Os sintomas se apresentam, na maioria dos casos, de forma tardia, sendo os principais as fístulas arteriovenosas e os pseudoaneurismas (PAs) 1 ^,^ 3 . 

 O manejo dessas lesões iatrogênicas apresentou grande avanço com o advento da cirurgia endovascular. Apesar de Inamasu et al. descreverem a excisão cirúrgica do PA com ou sem reconstrução arterial como principal tratamento, o complexo acesso cirúrgico pode levar a várias complicações 1 . 

 O presente artigo é um estudo comparativo de dois casos de lesões iatrogênicas semelhantes, pós-punção de acesso venoso profundo, mas com diagnósticos e abordagens diferentes. O objetivo é demonstrar que a evolução tecnológica impactou no tratamento do trauma vascular cervicotorácico de difícil acesso. Uma detalhada revisão da literatura contribui para o estudo da evolução desse tema e corrobora nossas conclusões. 

## DESCRIÇÃO DOS CASOS

 O primeiro caso, ocorrido em setembro de 1993, refere-se a uma paciente de 72 anos de idade, sexo feminino, portadora de cardiopatia grave, hipertensão arterial e obesidade grau III, internada em unidade de terapia intensiva com sepse de origem pulmonar. Foi submetida a punção de veia subclávia direita por técnica supraclavicular, sem sucesso. Uma semana depois, foi solicitado parecer à equipe de cirurgia vascular, sendo observada volumosa massa pulsátil na região cervical direita. Ao exame físico, a paciente apresentava-se hemodinamicamente instável, com uma volumosa massa pulsátil cervical direita ( Figura 1 ). O exame de *duplex scan* demonstrou a presença de PA oriundo da segunda porção da artéria subclávia direita (ASD), com 50 × 42 mm de diâmetro ( Figura 2 ), sendo indicada cirurgia direta. 

**Figura 1:** Massa cervical esquerda associada a hematoma.

**Figura 2:** Eco-Doppler demonstrando pseudoaneurisma com fluxo turbulento.

 A esternotomia mediana foi o acesso realizado, com extensão supraclavicular direita ( Figura 3 ). Procedeu-se ao isolamento da ASD proximal e progressivamente foi abordado o colo do PA. Este era originário do segmento proximal do tronco tireocervical, que foi simplesmente ligado, e o saco aneurismático foi esvaziado. A evolução foi satisfatória, apesar da magnitude da intervenção e da gravidade da paciente. 

**Figura 3:** Esternotomia com extensão supraclavicular direita.

 O segundo caso, ocorrido em dezembro de 2017, refere-se a uma paciente de 66 anos de idade, sexo feminino, portadora de cardiopatia grave e hipertensão arterial sistêmica, e submetida a transplante renal em 2007 devido a doença renal policística. Foi internada em unidade de terapia intensiva com sepse de origem pulmonar. Foi submetida a tentativa de punção de veia jugular interna esquerda ecoguiada para administração de aminas vasoativas; sem sucesso, o procedimento foi abandonado. A paciente evoluiu com massa pulsátil na região cervical esquerda, associada a queda progressiva do hematócrito. Após 15 dias de evolução com dor e expansão da massa cervical, foi solicitada avaliação da cirurgia vascular. 

 Ao exame físico e anamnese, a paciente queixou-se de dor importante na região cervical esquerda, associada a massa pulsátil ( Figura 4 ). O *duplex scan* sugeriu a presença de PA oriundo da artéria carótida comum esquerda ( Figura 5 ), e a angiotomografia computadorizada (ATC) demonstrou a presença de PA do segmento V1 da artéria vertebral esquerda, com 30 x 32 mm de diâmetro ( Figura 6 ). 

**Figura 4:** Massa cervical esquerda associada a hematoma.

**Figura 5:** Eco-Doppler demonstrando pseudoaneurisma (origem suspeitada ao nível da carótida comum esquerda – CCE), com fluxo turbulento.

**Figura 6:** Reconstrução 3D de angiotomografia da artéria vertebral esquerda, com o enchimento do pseudoaneurisma.

 Após discussão em equipe, optou-se pelo tratamento endovascular, com implante de um stent revestido Viabahn® 5 mm × 2,5 cm (WL Gore, Flagstaff, AZ, EUA) na artéria vertebral. O procedimento transcorreu sem intercorrências, sendo utilizados 18 mL de contraste iodado ( Figura 7 ). A paciente evoluiu com regressão da massa cervical e resolução da dor no pós-operatório imediato, sem déficit neurológico. *Duplex scan* de controle realizado após 6 meses do procedimento demonstrou perviedade da artéria vertebral esquerda ao nível do segmento V3. 

**Figura 7:** (A) Arteriografia peroperatória vertebral esquerda; (B) Arteriografia de controle pós-implante de Viabahn® 5 mm × 2,5 cm (WL Gore, Flagstaff, AZ, EUA).

## DISCUSSÃO

 As complicações vasculares relacionadas a procedimentos percutâneos na região cervicotorácica podem ser devastadoras 4 . Trauma iatrogênico em artérias carótidas e subclávias pode provocar sangramento intenso, hematomas, dissecção, embolia ou trombose, sendo descritos na literatura médica casos de obstrução de vias aéreas secundária a hematoma cervical em expansão, choque por hemotórax, acidente vascular cerebral devido a embolia, PA e fístula arteriovenosa 4 . O PA provavelmente é a complicação mais rara de lesão em artéria vertebral, com poucos casos descritos na literatura 3 ^,^ 5 ^-^ 8 e incidência de 0,2%, conforme reportado por Elias et al. 2 . 

 Os PAs da artéria vertebral podem ocorrer devido a trauma penetrante ou contuso da região cervical, dissecção arterial e após procedimento cirúrgico. Lesões penetrantes envolvem principalmente a primeira porção da artéria, antes da entrada pelo forame transverso, enquanto traumas contusos cervicais e cranianos acometem as porções mais distais 2 ^,^ 7 . A rápida identificação e conduta dessas lesões é primordial para um resultado satisfatório, apesar de o manejo ser pouco compreendido e raramente descrito na literatura 1 ^,^ 2 . Embora a resolução espontânea desses PAs ter sido descrita, rotura é observada em 31 a 54% dos pacientes 4 ^,^ 9 . 

 Os PAs se apresentam, na maioria dos casos, de forma tardia e principalmente sob a forma de massa pulsátil, com dor local, dispneia, insuficiência cardíaca e isquemia cerebral e medular 1 ^-^ 3 . Ambekar et al. descrevem diagnóstico após dois dias, Elias et al., após três dias, Bernik et al., após quatro dias, Amaral et al., após 15 dias, e Cihangiroglu, após 32 dias 2 ^,^ 3 ^,^ 5 ^,^ 7 ^,^ 10 . 

 A abordagem diagnóstica inicial descrita na literatura foi a realização de *duplex scan*, com visualização de hematoma com fluxo arterial em seu interior. Apesar de ser um bom método, a origem do PA pode não ser muito bem visualizada, conforme afirmam Cihangiroglu et al. 3 ^,^ 7 . A proximidade com a artéria carótida interna, o volume do hematoma e a turgescência da veia jugular interna são fatores que dificultam a visibilização de sua origem. Isso aconteceu nos dois casos relatados neste estudo. A ATC demonstra superioridade na visualização da lesão, incluindo sua origem, correta localização, presença de traumas vasculares adicionais e perviedade da artéria vertebral contralateral, além de auxiliar no planejamento terapêutico 2 ^,^ 3 ^,^ 7 . 

 O tratamento de PA inclui embolização endovascular, reparo direto da artéria vertebral ou ressecção do PA com ligadura 2 ^,^ 7 . Bernik et al. ainda inclui compressão guiada por ultrassom e injeção percutânea de trombina como opções alternativas 3 . Vale ressaltar que a profundidade da lesão impossibilita o tratamento compressivo e, pelo risco de embolização cerebral, o tratamento com trombina não é recomendado 3 . Aoki et al. descrevem o caso de um paciente tratado com conduta conservadora e que, após o 70º dia, evoluiu com dor e parestesia do ombro e braço direitos devido a compressão do plexo braquial pelo hematoma. O paciente evoluiu com rotura do PA, sendo operado em caráter de emergência com reparo direto; porém, foi a óbito no 11º dia de pós-operatório 8 . 

 Amaral et al. sugere o tratamento aberto como a primeira opção terapêutica quando a lesão é sintomática e localizada na origem da artéria vertebral 10 . A esternotomia mediana com extensão supra ou transclavicular é a melhor opção para controle proximal em vigência de grande hematoma à direita 3 ^,^ 7 ^,^ 10 . Outra opção de acesso é uma única incisão ao longo do músculo esternocleidomastóideo ou transclavicular, combinada com implante de balão temporário na origem da artéria vertebral 10 . Bernik et al. enfatizam a necessidade de um controle proximal ótimo para melhor visualização da lesão e tratamento cirúrgico 3 . À esquerda, uma abordagem envolveria toracotomia anterior para controle proximal da subclávia, pois a localização posterior dessa artéria não permite bom controle por esternotomia 11 . Todas essas abordagens envolvem morbidade elevada 11 ^,^ 12 . 

 Na literatura, o tratamento endovascular tem apresentado bons resultados 4 ^,^ 13 ^-^ 16 . Essa abordagem configurou uma mudança no manejo da lesão, que antes era realizado frequentemente por ligadura, para estratégias de exclusão do PA, mantendo a perviedade do vaso. Ambekar et al. e Kerolus et al. descrevem o uso de dispositivos de embolização como o Pipeline® (Covidien Vascular Therapies, Mansfield, MA, EUA), que promovem desvio de fluxo preservando a perviedade dos vasos. Em uma revisão sistemática, obtiveram uma taxa de obliteração aneurismática de 82,9% em 6 meses 5 . Pérez et al. utilizaram stent de cromo-cobalto balão-expansível revestido (Papyrus PK Biotronik®) para a exclusão de um PA da artéria vertebral, obtendo sucesso e mantendo a perviedade do vaso 13 . Kwon et al., além do stent revestido, também embolizaram o PA utilizando molas e obtiveram sucesso 16 . Yamamoto et al. descrevem o uso de molas e N-butil-2-cianoacrilato, com consequente oclusão da lesão, mas também da artéria vertebral 17 . Relatamos, provavelmente pela primeira vez, o uso de um stent revestido Viabahn® 5 mm × 2,5 cm (WL Gore, Flagstaff, AZ, EUA) na artéria vertebral, mantendo o vaso pérvio. 

 Assim como descrito por Amaral et al. e Yamamato et al., a oclusão unilateral da artéria vertebral é uma prática comum para o tratamento das lesões e, quando há perviedade da artéria contralateral, geralmente é bem tolerada 10 ^,^ 17 . A artéria cerebelar posteroinferior no lado da lesão deve ser suprida pela vertebral contralateral; se isso não ocorrer, pode-se desenvolver a síndrome de Wallenberg 10 . Essa síndrome é caracterizada por déficit nos nervos cranianos (V, IX, X e XI), ataxia, síndrome de Horner e perda contralateral da sensação de dor e temperatura, entre outras manifestações de insuficiência vertebrobasilar. É importante destacar que 15% da população tem uma artéria vertebral dominante, sendo a outra hipoplásica, e que 5% tem apenas uma artéria vertebral 10 . A oclusão ou ligadura da artéria vertebral em situações não emergenciais requer confirmação de fluxo sanguíneo adequado para a circulação posterior 10 ^,^ 18 ^,^ 19 . 

## CONCLUSÃO

 Embora usuais na medicina moderna, as punções venosas profundas não estão livres de complicações. O tratamento endovascular tem um importante papel na abordagem de pacientes com trauma vascular iatrogênico. Com isso, o papel do cirurgião vascular tradicional tem sido desafiado. Assim como na técnica de reparo aberto, o cirurgião vascular deve estar habituado com os avanços tecnológicos, tanto na propedêutica quanto na terapêutica. Com a técnica endovascular, geralmente há uma diminuição da morbidade e mortalidade no manejo de lesões de difícil acesso. 

 A introdução de novas tecnologias, como a punção ecoguiada, apesar de diminuir a ocorrência de complicações do procedimento, não evita iatrogenias. Apesar de treinamento e experiência, as punções venosas não estão livres de riscos. O conhecimento da anatomia cervical, bem como das suas variações anatômicas, faz-se necessário de modo tão relevante quanto o uso judicioso da ultrassonografia. A prevenção de lesões iatrogênicas é o principal ponto a ser ensinado nas instituições hospitalares 6 .
